# Decorrelation using optimal transport

**DOI:** 10.1140/epjc/s10052-024-12868-6

**Published:** 2024-06-06

**Authors:** Malte Algren, John Andrew Raine, Tobias Golling

**Affiliations:** DPNC, University of Geneva Faculty of Science, Geneva, Switzerland

## Abstract

Being able to decorrelate a feature space from protected attributes is an area of active research and study in ethics, fairness, and also natural sciences. We introduce a novel decorrelation method using Convex Neural Optimal Transport Solvers (Cnots) that is able to decorrelate a continuous feature space against protected attributes with optimal transport. We demonstrate how well it performs in the context of jet classification in high energy physics, where classifier scores are desired to be decorrelated from the mass of a jet. The decorrelation achieved in binary classification approaches the levels achieved by the state-of-the-art using conditional normalising flows. When moving to multiclass outputs the optimal transport approach performs significantly better than the state-of-the-art, suggesting substantial gains at decorrelating multidimensional feature spaces.

## Introduction

AI-powered decision-making has become a large part of automated systems in banks, advertising, and healthcare, to name a few. This has resulted in increased awareness surrounding the fairness and biases of the decision models. Due to the nature of many datasets, biases towards protected attributes like gender and race in data result in biased models. These biases are not only causes for concern in terms of fairness and ethics, but are also relevant to research in natural sciences, where correlations to protected variables are fundamental in nature, but can lead to undesirable effects in statistical analyses.

In High Energy Physics (HEP), classifiers are commonly used to separate different signal processes from background processes, in both event classification as well as object identification. One area which has seen a great deal of development is jet tagging, in particular identifying top quark initiated jets from the dominant QCD background of light quarks and gluons (see Ref. [1] for a comprehensive comparison of techniques). Identifying the origin of jets is not restricted to supervised classification, with anomaly detection being another area of active development in the hunt for physics beyond the standard model [2]. Here unsupervised or semisupervised classifiers are used to identify jets which may originate from new physics particles of unknown mass.

In the case of jet tagging, the desired accuracy of the classifier should be independent of the invariant mass of the jet, and instead exploit the differences in the underlying structure of the jet, known as jet “substructure”. However, while background processes often follow an exponentially decaying invariant mass distribution, the invariant mass of the signal processes is localised within some region of the mass spectrum. This overdensity of signal on the mass spectrum and correlations between the substructure of a jet and its invariant mass will lead to the classifier scores being correlated to the invariant mass. Several techniques have been developed which aim to decorrelate the scores of a classifier from the invariant mass of a jet [3–5]. These include methods that are employed during training as a means of regularisation, as well as post-training corrections.

In this work we introduce a new method for decorrelation using Convex Neural Optimal Transport Solvers (Cnots). Inspired by Ref. [5], which uses normalising flows to learn the monotonic transformation $T(\cdot |c)$ in 1D, given some protected attributes *c*, we propose to use the gradient of a convex neural network [6] for $T(\cdot |c)$ that by definition is monotonic in $\mathbb {R}^N$. We follow the case of conditional optimal transport studied in Refs. [7, 8]. We use convex neural networks to solve the Kantorovich dual formulation and find the optimal transport (OT) [9] between correlated scores and decorrelated ones. This function is a monotonic transformation $T(\cdot |c)$ between the two spaces that minimises the Wasserstein distance between them, and can therefore be applied to the input distribution as a means of decorrelation. We demonstrate the performance of this technique by applying it to jet tagging in HEP, where it is used to decorrelate both a binary classification discriminant and a three dimensional multi-class classifier output from the invariant mass of the jet.

## Methods

Decorrelation techniques are used in high energy physics for tasks like jet identification. These techniques address the issue of discriminators being strongly correlated to the jet mass due to varying mass values of resonant particles. The correlation is evident when visualizing discriminator scores as a function of mass. Some decorrelation methods modify the selection value for different mass values to maintain a fixed efficiency. However, we propose changing the discriminator score distribution to be invariant with respect to mass and to decorrelate the scores for any chosen efficiencies. This transformation should be a function of the mass.

OT methods represent a family of approaches where the transport function between two distributions can be learned, optionally conditional transport. OT methods are well-suited for decorrelation tasks. They can find a transport map between the correlated scores and a distribution that is the same for all mass value as it is inclusively, thereby achieving decorrelation. This approach not only provides a transport map which decorrelates the discriminant, but also uses the optimality constraint as a regularisation measure. This ensures stable and predictable behaviour across the entire discriminant space.

In an unconstrained transport function, the data order might change between spaces due to transport. This is undesirable when the spaces represent probabilities because it could lead to misinterpretations of the data. However, an OT function should be monotonic, preserving the data order. Any data swaps would incur additional transport costs, leading to suboptimal transport.

Monotonicity can be implemented in one-dimensional distributions easily, but it is more challenging to control in higher dimensions. This is because the optimization for the OT function needs to be done simultaneously with the control of monotonicity, which increases the complexity of the task. To obtain our decorrelation function we solve the Kantorovich dual formulation of optimal transport between our initial distribution and a target distribution enforced to be decorrelated to the jet mass.

### Dual formulation of optimal transport

Although the primal optimal transport problem can be used to measure the optimal transport cost between two distributions, in order to learn the optimal transport function between two continuous densities we need to use the dual formulation. Let *P*(*y*, *c*) and *Q*(*x*, *c*) be two continuous densities, where *x* and *y* are coordinates in an $\mathbb {R}^N$ space which follows, different distributions correlated to some latent conditional property *c*. The property *c* can be continuous or discrete in an $\mathbb {R}^M$ space. The optimal transport between these two densities is an optimisation problem over possible transportation maps, *T*, where

1 $$\begin{aligned} T^* = \inf _{T: T_\#(Q)=P} \frac{1}{2}\mathbb {E}||x-T(x|c)||^2. \end{aligned}$$

Here, $T_\#$ represents possible transports from *Q* to *P*, $T^*$ is the optimal transport between *Q* to *P* and $\mathbb {E}$ is the expectation value over *Q*.

**Fig. 1:**
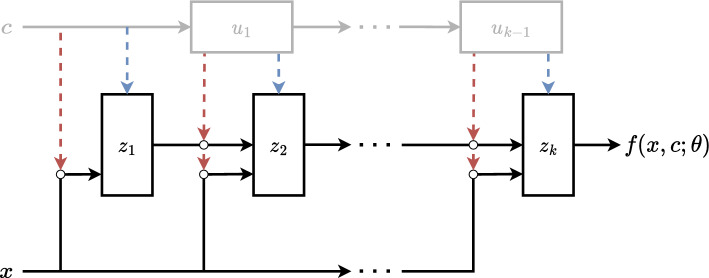
The architecture of the PICNN which parameterises a conditional convex function $f(x,c;\theta )$, that is convex in *x* but not in *c*. $\theta$ are the trainable parameters of the PICNN network. $\circ$ symbols indicate Hadamard products. The convex part of the network is indicated by the black lines and consists of recursive blocks $z_{1,\ldots ,k}$, that is defined in Eq. (4). The grey lines are the non-convex component consisting of $u_{1,\ldots ,(k-1)}$, defined in Eq. (3). The architecture reduces to an ICNN when removing the non-convex part

The problem can be formulated with a general cost or distance measure *d*(*x*, *y*), which here is chosen to be the squared Euclidean distance $d(x,y) = ||x-T(x)||^2$. For this cost function the optimal transport map is unique when *Q* is continuous [10].

It is possible to reformulate the primary problem in Eq. (1) as a dual formulation following Ref. [9] as

$$\begin{aligned} \mathbb {W}^2_2(P,Q) = \sup _{\begin{array}{c} f(y,c)+g(x,c)\\ \le \frac{1}{2} ||x-y||_2^2 \end{array}} \mathbb {E}(f(y,c)) + \mathbb {E}(g(x,c)), \end{aligned}$$

where $\mathbb {W}^2_2$ is the Wasserstein-2 distance. Here both *f* and *g* are functions constrained by

$$\begin{aligned} f(y,c)+g(x,c)\le \frac{1}{2} ||x-y||_2^2. \end{aligned}$$

By requiring *f* and *g* to be convex functions, this can be rewritten as

2 $$\begin{aligned} \mathbb {W}^2_2(P,Q)&= \mathcal {C}(x,y) + \sup _{\begin{array}{c} f(y,c)\\ \in \\ \text {cvx}(y) \end{array}}\,\mathop {\textrm{inf}}_{\begin{array}{c} g(x,c)\\ \in \\ \text {cvx}(x) \end{array}}\,\, f(\nabla g(x,c),c) \nonumber \\&\quad - \langle x,\nabla g(x,c) \rangle \nonumber \\&\quad - f(y,c), \end{aligned}$$

where $\mathcal {C}(x,y) = \frac{1}{2}(x^2+y^2)$ [9, 11]. Here both *f* and *g* are convex in *x* and *y*, respectively, but not in *c*. Under this formulation, the optimal transport map becomes

$$\begin{aligned} T_\#(x,c)=\nabla _x g(x,c)=P, \end{aligned}$$

the gradient of the convex function *g* with respect to its inputs *Q*, which, by definition, is monotonic in *x* for any given *c*.

This restriction assists the convergence towards the optimal transport map, which is also order preserving. One of the benefits of using $\mathbb {W}^2_2$ in comparison to divergences such as the Kullback–Leibler (KL) divergence is that it only requires samples from the input and base distributions, as opposed to the probability densities. Furthermore, it is well defined for all values whereas the KL divergence is not.

#### Convex neural networks

In Ref. [11], it is shown that Eq. (2) can be solved by parameterising the two convex functions with two Input-Convex neural networks (ICNN) [6] and thus learning the optimal transport as $\nabla _x g(Q;\theta )=P$ and $\nabla _y f(P;\theta )=Q$, where $\theta$ is the trainable parameters of the network. Partially input convex neural networks (PICNN) [6] extend ICNNs to take a conditional vector *c* in addition to the input vector *x* in order to represent a conditional convex scalar function $f(x,c;\theta )$. In this case $f(x,c;\theta )$ does not need to be convex with respect to *c*. The general architecture of the PICNNs can be seen in Fig. 1, whereby removing the non-convex part will reduce it to the ICNN.

Following the same notation as in Ref. [6], the PICNN is defined recursively in $z_k$ and $u_k$, where *k* is the number of layers ranging from $i=0,1,\ldots ,k$, and *W* and *b* are learnable parameters. The recursive non-convex layers follow a simple feed-forward network

3 $$\begin{aligned} u_{i+1} = \widetilde{g_i}(\widetilde{W}_i u_i+\widetilde{b}_i), \end{aligned}$$

where $u_0 = c$ and $\widetilde{g_i}$ is the activation function. These non-convex layers have no restrictions on allowed transformations and operations, as long as they are differentiable. The convex layers are defined as

4 $$\begin{aligned} z_{i+1}&= g_i \bigl ( W_{i, SP}^{z} \bigl ( z_i \circ [W_i^{zu}u_i+b^{z}]_{SP} \bigr )\nonumber \\&\quad + W_i^{y} \bigl ( y \circ (W_i^{yu} u_i + b_i^{y}) \bigr ) + W_i^u u_i + b_i \bigr ), \end{aligned}$$

where $z_0=0$. $W_{i, SP}^{z}$ are restricted to be strictly positive by passing the weights through a softplus transformation, and the activation functions $g_i$ are required to be convex and non-decreasing. Lastly, the output is defined as

5 $$\begin{aligned} f(x,c;\theta ) = \frac{w_{0, SP}(u_k)}{2}x^2 + w_{1, SP}(u_k) z_k, \end{aligned}$$

such that the transport function given by

6 $$\begin{aligned} \nabla _xf(x,c;\theta ) = w_{0, SP}(u_k) \cdot x + w_{1, SP}(u_k) \nabla _x z_k \end{aligned}$$

makes perturbations around the identity function easily accessible and the transport at initialisation for random weights $\theta$.

#### Convex neural networks for decorrelation

In order to use convex neural networks for decorrelation purposes, the aim is to learn a monotonic transformation $T(\cdot |c)$ between the input feature space $Q(\cdot |c)$ and a target feature space $P(\cdot )$. To break the dependence on *c*, we construct *P* to be a distribution which is identical for all values *c*. In this framework, the choice of *P* is completely free and does not need to follow an analytically defined function. This is in contrast to normalising flows, which require the probability distribution function of the base density to be analytically calculable. Alternatively, normalising flows can also use arbitrary PDFs as base distributions [12], though we do not study this here. The simplest way to construct $P(\cdot )$ is to take the distribution $Q(\cdot |c)$ and randomly permute the conditional vector, breaking the correlation. This should also define a target base distribution which is similar to the input distribution, simplifying the transport function. Other choices are to choose a uniform distribution or normal distribution, as is done for cflows in Ref. [5].

### Other exiting methods

#### Conditional normalising flows

Conditional normalising flows (cflow) [13, 14] are networks built to be fully invertible, therefore by definition *f* and $f^{-1}$ exist and are fast to compute. This makes cflows powerful as generative models using the change of variables formula

7 $$\begin{aligned} p_x(x|c) = p_y(f(x,c)) \left| \det \mathcal {J}(f(x,c)) \right| , \end{aligned}$$

to transform between base density $p_y(x)$ independent in *c* and some complex density $p_x(x|c)$ conditioned on *c*. The objective of the cflow will be to maximise the log-likelihood, which requires the knowledge of the PDF of the base density $p_y(x)$. By construction, the cflow can be inverted $f^{-1}(p_x(x|c))\rightarrow p_y(x)$ to be independent of *c*, producing the desired decorrelated features [5].

For $p_x(x|c)$ in 1D, the cflow transformation can be restricted to be monotone ensuring order preservation, which is important for decorrelating discriminate scores. However, beyond 1D, the monotonicity of the transformations is not guaranteed.

An invertible PICNN architecture can also be trained like a normalising flow to maximise log-likelihood [15] and find a monotonic transformation in $\mathbb {R}^N$. However, this involves calculating the Hessian in the forward pass, which is an expensive procedure and limits the choice of base distribution to analytically defined functions. Due to the additional complexity, this training scheme is not studied in this work.

#### Decorrelating during training

A wide range of established methods for the decorrelation of classifier outputs are applied before or during the training of the discriminator. Planing [16] can be applied to the data beforehand, as a form of preprocessing, ensuring that the distribution over the protected variables *c* follow the same distribution for both the signal and background jets. An alternative approach is penalising the classifier for producing outputs which are correlated with *c* during training by adding an additional loss or regularisation term $\mathcal {L}_{corr}$. Example methods calculate $\mathcal {L}_{corr}$ with adversarial neural networks [17–19], or with distance measures calculated using distance correlation [4] or the moments of the conditional cumulative distributions [3]. The total loss for these methods is given by

8 $$\begin{aligned} \mathcal {L} = \mathcal {L}_{class} (s(x), y)+\alpha \mathcal {L}_{corr}(s(x), c), \end{aligned}$$

*c* being the protected attributes, *y* are the labels and *s*(*x*) is the classifier output during training. The decorrelation can then be controlled by a hyperparameter $\alpha$.

## Application to jet tagging

In HEP, multiple studies have been conducted to decorrelate the predictions *s*(*x*|*m*) of binary classifiers from the invariant mass *m* of a reconstructed object [3–5, 16–20]. However, balancing the classification loss and decorrelation loss has proven difficult. The current state-of-the-art approach uses conditional flows and imposes no restrictions on either the dataset or the architecture of the classifier.

To form a basis of comparison and evaluate the performance between Cnots and cflows, we look at decorrelation of classifiers trained to identify the origin of boosted objects at particle colliders like the LHC [21]. When collisions produce particles with high transverse momentum, their decay products have a smaller opening angle. In the case where these particles decay to partons, the two resulting hadronic showers, known as jets, start to overlap. In this instance, their decay products are unable to be resolved individually and instead are reconstructed as a single large jet. Differences in the underlying structure within the jet can be exploited to predict the initial particle produced in collisions. However, the underlying structure of jets remains strongly correlated to the reconstructed invariant mass of the jet resulting in a biased prediction of the initial particle. In this work we study the performance of decorrelating classifiers trained to identify boosted jets origination from quarks and gluons (QCD), vector bosons (VB) and top quarks (Top), from the invariant mass of the jet.

### Datasets

To get pure samples of the top quark initiated jets, samples of $t\bar{t}$ jets are produced, in which both the top quarks decay hadronically. For a pure sample of vector boson initiated jets, samples of *WZ* diboson events are generated in which both the *W* and *Z* bosons decay to two quarks. For pure samples of QCD initiated jets, samples of two-to-two processes with a final state of two quarks and or gluons are simulated. All three samples are generated at a centre of mass energy $\sqrt{s}=$ 13 TeV using MadGraph_aMC@NLO [22] (v3.1.0), with decays of top quarks and *W* bosons modelled with MadSpin [23]. Pythia8 [24] (v8.243) is used to model the parton shower and hadronisation with the NNPDF2.3LO PDF set [25]. The detector response is simulated using Delphes [26] (v3.4.2) with a parametrisation similar to the ATLAS detector [27]. Jets are reclustered using the anti-$k_t$ clustering algorithm [28] with a radius parameter $R=1.0$ using the FastJet package [29]. Jets are required to have $p_\textrm{T}>450$ GeV and $|\eta |<2.5$, with only the jet with the highest $p_\textrm{T}$ selected from each event. The minimum $p_\textrm{T}$ of the leading parton in the hard scatter is optimised for each sample in order to increase the rate of jets passing the selection criteria and in order to produce similar distributions for the jet $p_\textrm{T}$ across all three jet types.

The relative four momenta ($p_\textrm{T}^{\text {frac}}$, $\Delta \eta$, $\Delta \phi$, $E^\text {frac}$) of up to the leading 100 constituents, ordered in descending $p_\textrm{T}$, are stored for each jet, alongside the jet four-momentum vector ($p_\textrm{T}$, $\eta$, $\phi$, *m*). Jets with fewer than 100 constituents are zero-padded. In total just under 4 million QCD, 2 million VB and 2 million Top jets are generated, with independent selections for training the classifiers, the decorrelation approach and performance evaluation.

The classifiers are trained using 840,000 jets each of QCD, VB and Top, including the validation set. Both decorrelation methods are trained with the same two million QCD events, with jets required to have an invariant mass between 20 and 450 GeV. Performance is evaluated with independent test sets comprising 1 million jets each of QCD, VB and Top.

### Classifiers

A multiclass classifier (mDNN) is trained to predict the probabilities of a jet originating from each jet type, $p_{QCD}, p_{T}$ and $p_{VB}$. The mDNN is constructed using the Particle-Transformer architecture from Ref. [30], and is trained using the constituents of the jets.

Whilst the multi dimensional scores from the mDNN are correlated to mass, visualising the correlated scores in $\mathbb {R}^3$ is difficult. Thus, for visualisation purposes, we project the 3D scores down to 1D distributions following the Neyman Peason lemma to create three discriminators $\mathcal {D}$

9 $$\begin{aligned} \mathcal {D}_\textrm{QCD}&= \frac{p_{QCD}}{p_{T}+p_{VB}},\nonumber \\ \mathcal {D}_\textrm{T}&= \frac{p_{T}}{p_{QCD}+p_{VB}},\nonumber \\ \mathcal {D}_\textrm{VB}&= \frac{p_{VB}}{p_{T}+p_{QCD}}. \end{aligned}$$

To evaluate the performance of decorrelating the output of a binary classifier, we use the discriminator scores $\mathcal {D}_\textrm{VB}$ normalised with a sigmoid transformation. For the decorrelation of a multiclass output, we decorrelate the joint distribution $p_{QCD}, p_{T}$ and $p_{VB}$. In the 3D case, the discriminators in Eq. (9) are used for visualisation.

**Fig. 2:**
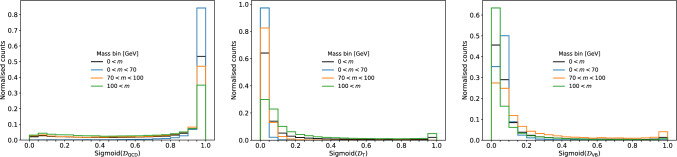
The scores from the Transformer projected down to 1D discriminators using Eq. (9) with additional sigmoid applied for normalisation. The figures only comprises of QCD events

**Fig. 3:**
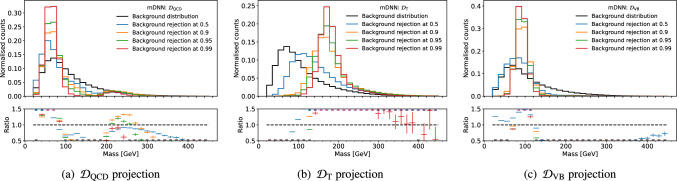
Mass distribution at background rejections of 50%, 90%, 95% and 99% for the raw discriminators. We always consider QCD the background distribution

The three discriminators are shown in Fig. 2, where we can see that the scores change as a function of mass. The mass sculpting is also very apparent when applying a selection on the discriminate scores, as seen in Fig. 3, where, especially for the $\mathcal {D}_\textrm{VB}$ and $\mathcal {D}_\textrm{T}$, the sculpting surrounding the resonance mass is evident.

## Results

While the decorrelation methods are trained exclusively on QCD jets to ensure that there is no underlying correlation between classes, evaluation includes all classes.

To evaluate the decorrelation performance, prior decorrelation studies in HEP have used the inverse Jensen-Shannon divergence (1/JSD) between the initial invariant mass distribution and the distribution after applying a selection on the classifier scores. This is an effective measure of the mass sculpting resulting from the classifier output. In the context of HEP analyses, we look at a board range of background rejections, not just individual values, as these are task specific [18, 31, 32]. It is desirable minimize sculpting in order not to need to worry about calibrating background response in downstream task. As the decorrelation methods are trained on QCD jets, fully decorrelated classifier scores should not sculpt the initial mass distribution after a selection. In order to estimate an upper bound on performance arising from the statistical variation of the data, we calculate the ideal 1/JSD using 32 bootstraps [33] on the initial QCD mass distribution without a selection. To measure the discrimination power after decorrelation, we calculate the signal efficiency for both the Top and VB as a function of background rejection, and calculate how the area under the receiver operating characteristic curve (AUC) changes as a function of mass.

We find that training of the two PICNNs to decorrelation the distribution is very stable and despite only being limited to convex function, it is very expressive with a low number of learnable parameters.

Given that both the Cnots and cflows decorrelation approaches are post-processing methods, and the state-of-the-art performance observed with cflows, we limit our comparisons to these two. Other existing decorrelation methods could potentially be integrated with either Cnots or cflows. As demonstrated in Ref. [5] for cflows, employing post-processing decorrelation aids in decorrelating the correlated distribution.

### Binary decorrelation

**Fig. 4:**
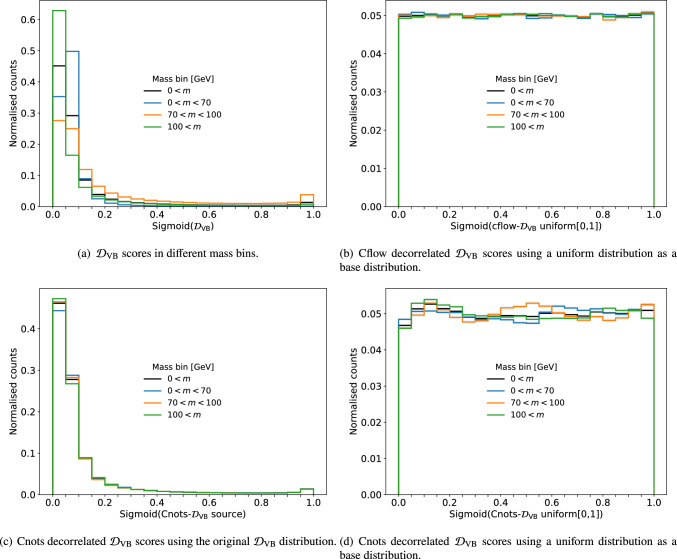
The $\mathcal {D}_\textrm{VB}$ scores in different mass bins. Sigmoid is applied to normalise between [0,1]. **a** Shows the original distribution. In **b**–**d**, the scores are decorrelated to different base distributions using the decorrelation methods explained in Sect. 2

The correlated $\mathcal {D}_\textrm{VB}$ scores are shown in Fig. 4 as a function of mass, where a dependency on mass is apparent, especially around the W/Z-boson mass.

In Fig. 4, we trained the decorrelation methods explained in Sect. 2 and applied the learned transformation to decorrelate the $\mathcal {D}_\textrm{VB}$ scores. For both methods, the mass dependency is removed and the distribution of the scores are the same in the four mass bins. The Cnots method for 1D decorrelation of $\mathcal {D}_\textrm{VB}$ (Cnots-$\mathcal {D}_\textrm{VB}$) is able to decorrelate to an arbitrary base distribution, whereas the cflow method has to evaluate the likelihood of the PDFs. Therefore, we show two possible base distributions for Cnots, one using a uniform distribution, and another using the source distribution as the base distribution.

**Fig. 5:**
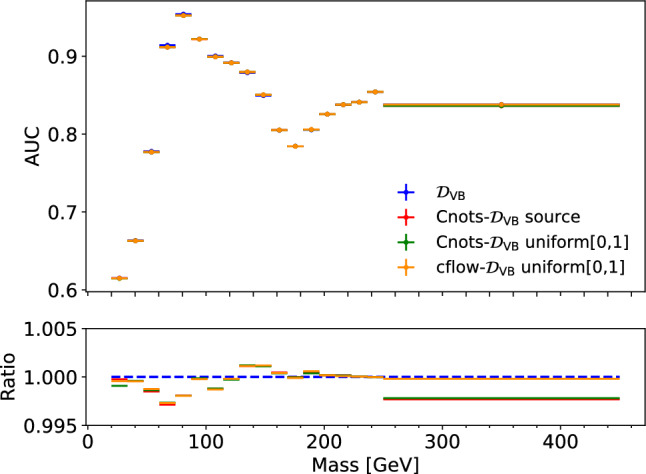
Comparison between the AUC before and after decorrelation as a function of mass. The ratio is calculated between the binned AUC values of the decorrelated $\mathcal {D}_\textrm{VB}$ and the initial discriminator

After decorrelation, the inclusive separation power will degrade, as $\mathcal {D}_\textrm{VB}$ scores are not able to discriminate using the additional separation power arising from the jet mass. However, due to the monotonicity of the transformations, the integrated performance over the protected attributes should remain the same. In Fig. 5, we select jets within narrow mass bins to imitate the integration and calculate the AUC. We see here that the AUC as a function of mass remains unchanged after decorrelation.

**Fig. 6:**
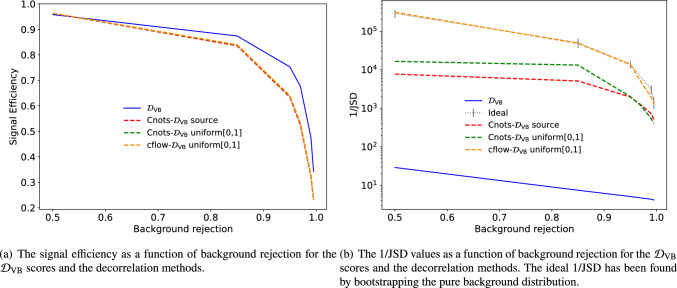
The signal efficiency and 1/JSD are measured at various levels of background rejection. **a** Shows the signal efficiency at the different background rejections. **b** Shows the background sculpting at the different background rejections. 1/JSD is measured only on QCD jets

To evaluate the decorrelation performance, we measure the 1/JSD at different selections and simultaneously measure the signal efficiency of VB. This is illustrated in Fig. 6 for the $\mathcal {D}_\textrm{VB}$, cflow-$\mathcal {D}_\textrm{VB}$ and Cnots-$\mathcal {D}_\textrm{VB}$. While we see no large variation in the signal efficiency between the decorrelation, the cflow method outperforms the Cnots methods in the background sculpting.

### Multiclass decorrelation

To assess the decorrelation performance of the methods, we look at the background sculpting and signal efficiency of the discriminators after applying decorrelation.

We test Cnots applied to the classifier outputs with two different base distributions. Cnots Dir(1,1,1) uses 3D logit-Dirichlet as a base distribution with the concentration parameters set to one, Cnots source uses the original mDNN scores as a base distribution, and cflow uses a normal distribution as a base distribution. All methods are trained in logit space and normalised with softmax during evaluation. A comparison of Cnots and cflow using the same base distribution is made in Appendix E.

**Fig. 7:**
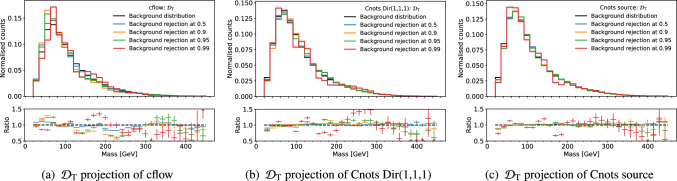
Mass distribution at background rejections of 50%, 90%, 95% and 99% on $\mathcal {D}_\textrm{T}$ after decorrelating using three methods

In Fig. 7, we see an example of mass sculpting at different background rejections on $\mathcal {D}_\textrm{T}$. Comparing to the original sculpting in Fig. 3, the methods have all reduced the amount of sculpting, however, we see more fluctuations present in cflow than its Cnots counterpart.

**Fig. 8:**
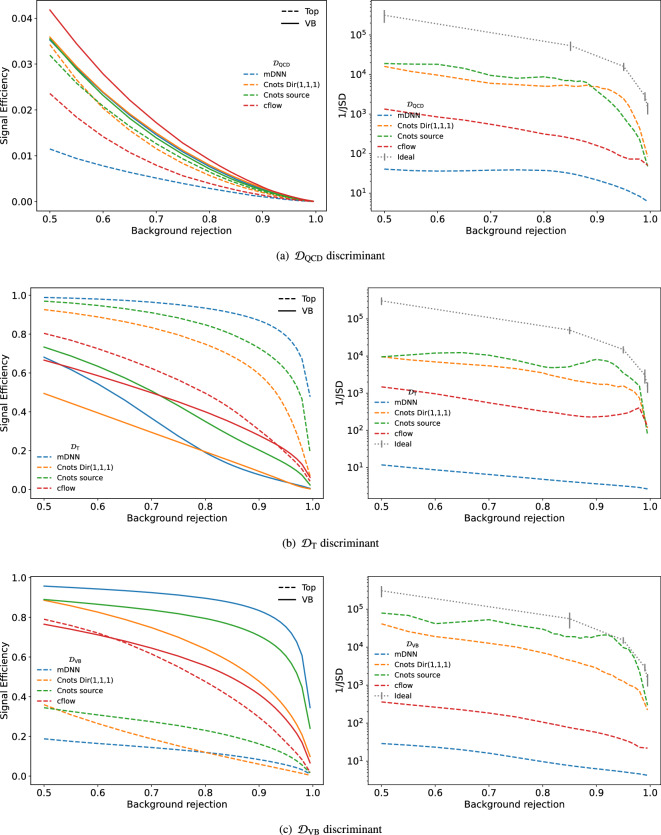
The signal efficiency and 1/JSD are measured at various levels of background rejections with the different discriminators from Eq. (9). The left column shows the signal efficiency. As we only consider Top and VB as signal, only the Top and VB signal efficiency are shown. The right column shows the measured background sculpting for different selections. The 1/JSD is measured only on QCD jets

**Fig. 9:**
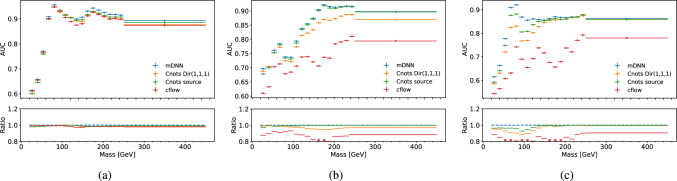
Comparison between the AUC before and after decorrelation as a function of mass for **a** $\mathcal {D}_\textrm{QCD}$, **b** $\mathcal {D}_\textrm{Top}$ and **c** $\mathcal {D}_\textrm{VB}$. The ratio is calculated between the original AUC and the decorrelated ones

To quantify the difference in performance, we use 1/JSD and the signal efficiency seen in Fig. 8. We see both Cnots models outperform the cflow for all three discriminants. We also see that, in addition to the reduced levels of sculpting, the Cnots models consistently have a higher signal efficiency than cflow for the discriminator optimised for the target jet type. The poor decorrelation performance of the cflow lies in its unconstrained transport maps, which do not contain the restriction that the map must be monotone and therefore allows order swapping. However, this is not the case for Cnots, which is order preserving by construction. This is also clear from the differences in AUC as a function of jet mass shown in Fig. 9, where the integrated performance of cflow declines significantly compared to the original distribution. We also see that the performance for the two Cnots methods is close to the optimal performance of the original distribution. The small discrepancy may be due to the finite binning size of the integral.

#### Order preservation

The measure of order preservation is simple to quantify in 1D, however, it becomes non-trivial in higher dimensions. For an order preserving map, we expect to observe no rapid fluctuation in the transport map and minimum curl in the transport field. We attempt to visualise the level of order preservation by depicting the gradient of the transport maps across the input space. Low and smoothly changing gradients indicate a smooth and order preserving map. However, large gradients or abrupt changes do not necessarily indicate where the order preservation is broken.

In order to compare the transport maps between Cnots and cflow, a shared base distribution is required. We choose a normal distribution as the base distribution for both. In order to reintroduce unitarity per event, the outputs are rescaled using a softmax activation on the three outputs, ensuring two degrees of freedom. We then visualise the transport maps in 2D by using $p_{VB}$ and $p_{T}$.

**Fig. 10:**
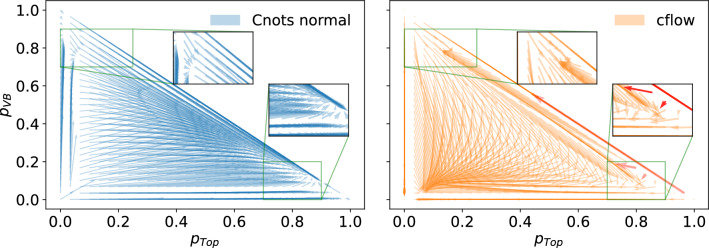
By taking the difference between points before and after decorrelation, we can calculate the displacement vector and construct the transport maps for both methods. These displacement vectors are visualised in this quiver plot. The transport maps are mass dependent, so to compare maps, the same mass value has to be chosen. For these two specific transport maps, the W-boson mass is chosen. The red arrows are the cflow displacement vectors that are above the maximum magnitude differences of the Cnots seen in Fig. 11

**Fig. 11:**
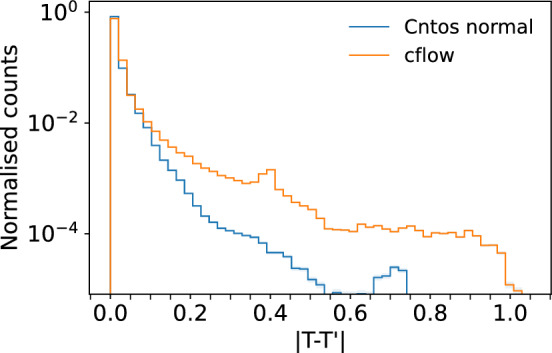
The magnitude of the difference between the original displacement vectors and their small deviation counterpart

Taking the difference between correlated and decorrelated scores, we can calculate a displacement vector that indicates the direction and amount a given point is transported during decorrelation. As the scores are in Dirichlet space, one of the three dimensions becomes redundant and can be dropped, enabling us to show the transport maps in 2D. The displacement vectors are shown in Fig. 10 for Cnots and cflow. We show that in some regions the transport map of the cflow changes rapidly and overlaps into other regions, whereas the Cnots has smoother transitions. We will attempt to outline these rapid transitions by sampling random positional variations of $2\%$ deviation from the original points in Fig. 10. We then measure the distance between displacement vectors of the original point and a small deviation. A large difference indicates a rapid change in the transport map, which can result in non-order preserving transport. In Fig. 11, the histogram of magnitudes is shown. Here, $1 \%$ of the cflow displacement vectors have a magnitude larger than the maximum magnitude of the Cnots. These displacement vectors are indicated in red in Fig. 10. These red arrows strongly indicate that we have regions in the cflow transport map where the monotonicity is broken, which results in the low signal efficiency we saw in Fig. 8.

**Fig. 12:**
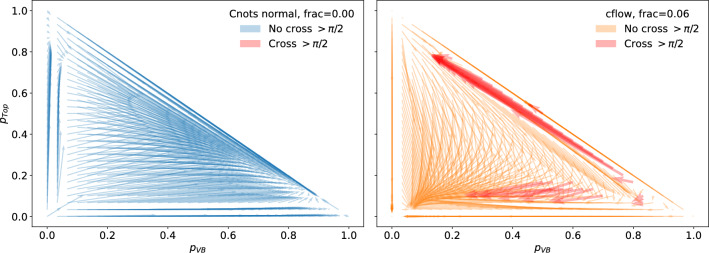
The displacement vectors for the transport maps of the Cnots (left) and cflow (right). An invariant mass of 80.377 [GeV] is selected for these transport maps. Displacement vectors that cross each other by an angle greater than $\pi /2$ are highlighted in red, highlighting where the transport is non-convex

As another measure of order preservation, we try to quantify areas of the transport map where order swapping may occur. In the case of a perfectly monotonic transport in two dimensions, the transport field should be convex, we postulate that for this to hold no transport paths should cross with an angle greater than $\pi /2$. In Fig. 12, we show the same transport maps as in Fig. 10, but highlight transport vectors that cross any other transport vector by an angle greater than $\pi /2$. Here we see that this does not hold for cflows, however, it does hold for Cnots.

These rapid changes in the transport map will break the monotonicity. However, a clear quantification of monotonicity in higher dimensions are not known to us.

## Conclusion

In this work we have introduced a novel method for decorrelating feature spaces correlated to protected attributes by finding the optimal transport map between the correlated space and a decorrelated space. This map is constructed as the gradient of a partially input convex neural network, ensuring it is monotonic by construction. We study the decorrelation performance of our approach in comparison to the state-of-the-art for jet tagging at the LHC. Conditional normalising flows [5] have demonstrated success in decorrelating 1D distributions, with our approach reaching similar levels of performance. However, Cnots achieves state-of-the-art performance outperforming the normalising flows at decorrelating higher dimensional feature spaces. This increase in performance is achieved due to the enforced monotonicity in the architecture, which, although present in 1D for the normalising flows, is not enforced in higher dimensions. Furthermore, Cnots can perform decorrelation with an arbitrary distribution chosen as the target of the transport.

The application of decorrelation is not restricted to classifier outputs, and due to the state-of-the-art performance in decorrelating higher dimensional feature spaces, Cnots should result in improved performance for tasks which require decorrelation of input feature spaces.

## Figures and Tables

**Fig. 13:**
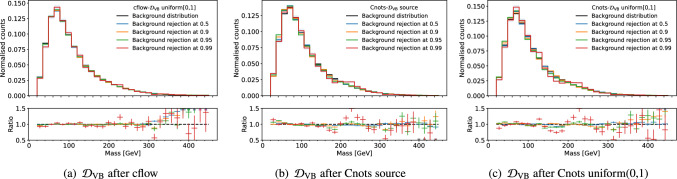
The figures show the mass sculpting given a background rejection for the different decorrelation methods

**Fig. 14:**
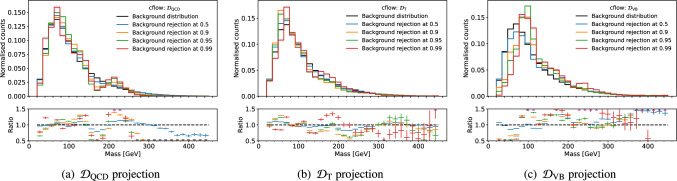
Mass distribution at background rejections of 50%, 90%, 95% and 99% after decorrelating the mDNN scores using the cflow method

**Fig. 15:**
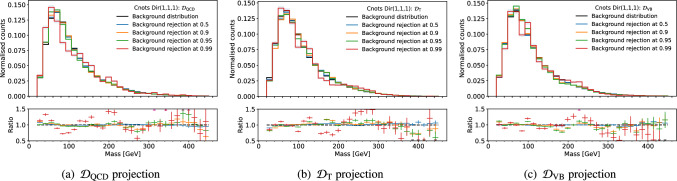
Mass distribution at background rejections of 50%, 90%, 95% and 99% after decorrelating the mDNN scores using the Cnots method with logit-Dirichlet as a base distribution

**Fig. 16:**
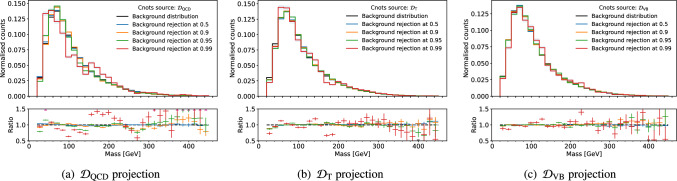
Mass distribution at background rejections of 50%, 90%, 95% and 99% after decorrelating the mDNN scores using the Cnots method with the original source distribution as a base distribution

**Fig. 17:**
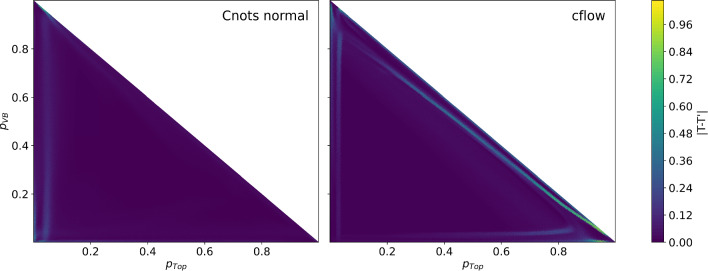
Taking the displacement vectors from Fig. 10 and calculate the same vectors with a positional uniform variation of $1\%$, we can measure the magnitude change of the transport map. The colour indicates the change scaled with *log*

**Fig. 18:**
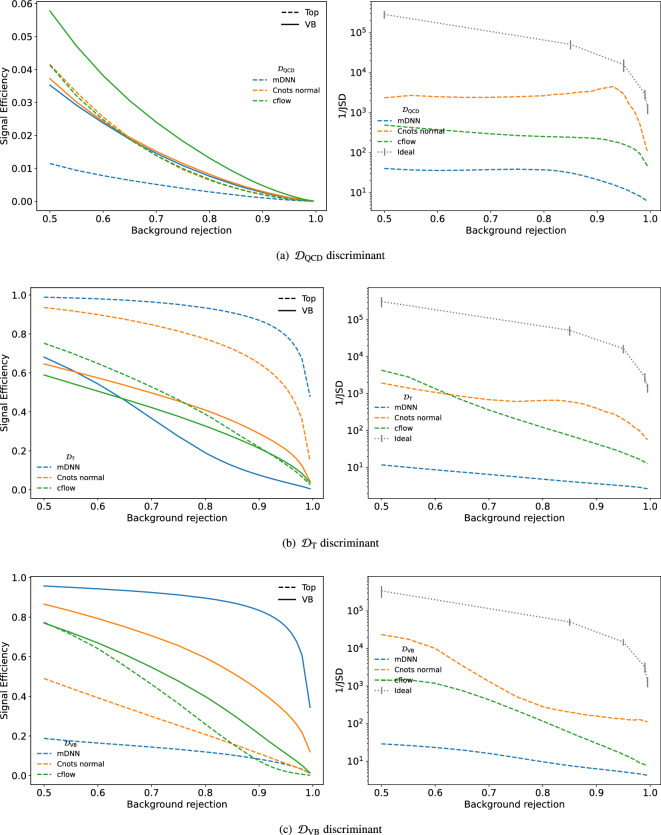
The signal efficiency and 1/JSD are measured at various levels of background rejections with the different discriminators from Eq. (9). The left column shows the signal efficiency. As we only consider Top and VB as signal, only the Top and VB signal efficiency are shown. The right column shows the measured background sculpting for different selections. The 1/JSD is measured only on QCD jets

**Fig. 19:**
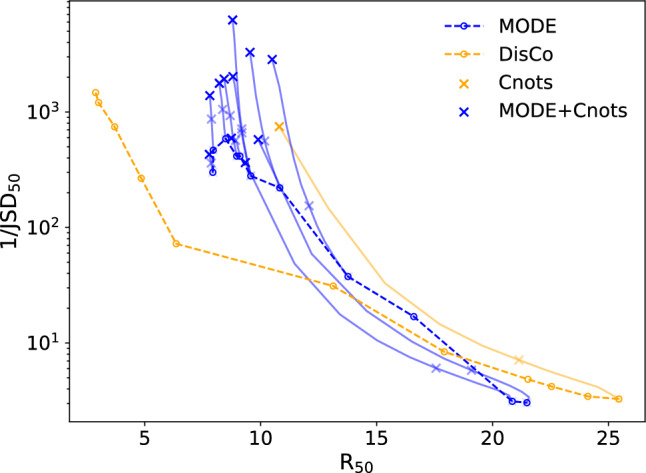
Performance of the different decorrelation methods on the Boosted W dataset. The rejection factor is the inverse of the false positive rate at a 50% true positive rate. The $1/JSD_{50}$ is measured at each rejection factor. The lower the $1/JSD_{50}$, the better the decorrelation. The dashed lines use the decorrelation methods from Refs. [3, 4] and the solid lines show the decorrelation trajectory calculated by Cnots on each MODE point

## Data Availability

Data will be made available on reasonable request. [Author’s comment: The datasets generated during the current study are available from the corresponding author upon reasonable request.]

## Multiclass classifier

The transformer use 3 self-attention layers and 2 cross-attention layers, each with an embedding dimension of 128 and 16 attention heads. The 7 node features and 5 edge features are embedded into this space each using a dense network with a single hidden layer of 256 neurons. We also use the same dense network shape for the residual updates inside the transformer, and to extract the final 3-value discriminant from the cross attention blocks. Conditioning on the 2 high level variables is achieved by concatenating them to the input of all dense networks use in this setup. We use the LeakyReLU activation and a dropout probability of 0.1 in all linear layers. We trained the transformer for 10 epochs using the AdamW optimiser with a learning rate of 0.001 and a weight decay strength of 0.0001.

## Cflow

The cflow for binary decorrelation is training using Adam optimiser for 300 epochs with initial learning rate at 0.0005 that annealed to zero using cosine annealing with batch size of 512. The cflows is constructed by 5 rational quadratic spline layers with 12 bins in each layer using a uniform[0,1] or a normal distribution as a base distribution for either binary and multiclass decorrelation, respectively. The invariant mass is transformed to logarithmic mass. The multiclass decorrelation cflow is trained with same training setup as the binary one. However it is constructed by 6 rational quadratic spline layers with 12 bins bounded within [$-$3.5, 3.5] in each layer using a normal distribution as a base distribution. Outside the bins, the map is linear. Everything else followed the default settings from Ref. [34].

## PICNN

**Table 1:** The table show the hyperparameter for the *f* and *g* network use for our decorrelation method. Zeroed softplus is $SP(x)-SP(0)$

Category	Hyperparameter	Value
Training	Optimiser	AdamW(0, 0.9)
	Epochs	150
	F per g	4
	G per f	10
	Gradient clipping	5
	Number of batches per epoch	512
	Batch size	1024
	Learning rate	0.001
	Scheduler	CosineAnnealingLR
Network	Convex layer size	64
	Non-convex layer size	8
	Number of layers	4
	Convex activation function	Zeroed softplus
	Nonconvex activation function	Elu

The architecture for the PICNNs use in both binary and multiclass decorrelation can be seen in Table. 1. We always train our transport in logit space with logarithmic invariant mass as conditions. For both multiclass and binary decorrelation we test the same two base distributions, a uniform[0,1] and the original source distribution.

## Binary decorrelation

To compare the 1/JSD values for the different methods, the binning and ranges between distribution has to be the same. We have chosen 50 bins equally spaced between 20 and 450 GeV for all mass sculpting figures. In Fig. 13, we see the mass sculpting at different background rejections. These are the distribution that are used to measure the 1/JSD values. We see that after decorrelating the scores of $\mathcal {D}_\textrm{VB}$, the sculpting becomes non-existent.

## Multiclass decorrelation

In Figs. 14, 15 and 16, we see the mass sculpting after using the decorrelation methods. Comparing to Fig. 3, where we see the original mass sculpting for all three discriminators, we now see that sculpting has been reduce, which is also clear from Fig. 8.

Cnots normal has a smooth change in magnitudes indicated by the smooth color transitions in Fig. 17. However, there are artifacts within magnitude difference of the cflow, meaning rapid changes in the transport map. These will break the monotonicity, which can also be seen from the overlapping displacement vectors from Fig. 10.

The decorrelation performance of the model, used in Sect. 4.2, is illustrated in Fig. 18. In this case, both Cnots and cflow share the same base distribution. When using the normal distribution as a base, we observe that the performance of Cnots is generally better (considering both signal efficiency and 1/JSD). However, it falls short when compared to the performance achieved with other base distributions mentioned in Sect. 4.2.

## Postprocessing of existing decorrelation methods

Previous HEP decorrelation studies[3–5] have utilised the dataset generated in Ref. [4] to study binary decorrelation performance. This dataset consists of Boosted W and QCD, which makes it suitable for only the binary case.

To evaluate the postprocessing capabilities of Cnots, we train the decorrelation methods described in Refs. [3, 4] and afterwards apply Cnots to further improve decorrelation. We will be using the postprocessing capabilities of Cnots on MODE as it has been shown to have the best decorrelation performance and will only use Cnots on the vanilla DisCo classifier trained using binary cross entropy.

The classifiers will be trained with varying decorrelation strengths $\lambda$. Technically, Cnots calculates a displacement vector for each event, which can be used to map out the trajectory of the decorrelation. Thereby, we can control the decorrelation strength of Cnots by choosing different trajectory points along the OT.

We will be using the metrics established in Refs. [3, 4], where the decorrelation performance is measured using the rejection factor ($R_{50}$), which is the inverse of the false positive rate at a 50% true positive rate, where at each rejection factor the $1/JSD_{50}$ is measured.

The performance of the different decorrelation methods can be seen in Fig. 19. We see that Cnots is able to further improve the decorrelation performance of MODE by increasing the $1/JSD_{50}$ without significantly lowering the $R_{50}$. We can also use the trajectory of Cnots to choose a decorrelation point.

## Code Availability Statement

This manuscript has associated code/software in a data repository. [Author’s comment: The code is available at https://github.com/malteal/ot-decorrelation.]
